# Improving Ramie Fiber: Current Progress and Future Directions in Molecular Breeding

**DOI:** 10.3390/plants15101435

**Published:** 2026-05-08

**Authors:** Linfeng Su, Fang Liu, Yinghong Tang, Song Gao, Hangfan Niu, Yanzhou Wang, Jianrong Chen, Touming Liu

**Affiliations:** 1 College of Biological and Chemical Engineering, Changsha University, Changsha 410022, China; feo.sulf@gmail.com (L.S.); lf800825@163.com (F.L.); 2 College of Life and Environmental Science, Hunan University of Arts and Science, Changde 415000, China; yinghongtang2019@163.com; 3 College of Horticulture and Landscape Architecture, Yangzhou University, Yangzhou 225009, China; gaosong@yzu.edu.cn (S.G.); 17651976225@163.com (H.N.); 4 Institute of Bast Fiber Crops, Chinese Academy of Agricultural Sciences, Changsha 410205, China; wyzhcf@163.com

**Keywords:** ramie (*Boehmeria nivea*), fiber quality, degumming, secondary cell wall, genetic transformation, molecular breeding

## Abstract

Ramie (*Boehmeria nivea*) is renowned for its superior fiber strength, length, and unique properties, yet its genetic improvement has lagged behind other fiber crops. This review synthesizes recent advances in ramie fiber development at the genetic, genomic, and molecular levels. Population genomic analyses have uncovered distinct domestication, improvement, and feralization signatures, identifying numerous fiber-related genes under selection. Parallel genetic and molecular studies have mapped scores of loci and genes governing fiber formation, laying the foundation for molecular breeding. However, despite the availability of genetic transformation systems, the need for methodological improvements remains a major challenge for engineering fiber traits via transgenic approaches. Overall, a solid research foundation has been established. Future progress in establishing marker-assisted and genomic selection breeding systems, optimizing transformation protocols, and developing efficient gene-editing methods holds promise for realizing molecular breeding to enhance fiber quality and yield in ramie.

## 1. Introduction

Ramie (*Boehmeria nivea*) is an important natural bast fiber crop whose exceptional mechanical properties distinguish it from most other fiber plants. In addition to its high strength, ramie exhibits excellent moisture absorption, antibacterial activity, and biocompatibility, enabling its use not only in traditional textile industries but also in composite materials, biomedical applications, and other specialized fields [1,2,3,4]. Historically, ramie has been utilized for several millennia [5] and has developed a solid industrial foundation. In 2018, the production value of ramie was approximately USD 0.1 billion, higher than that of hemp and flax fibers, although its production volume (100,000 tons) was lower than that of flax (310,000 tons) [6]. Building on this industrial foundation, the growing demand for natural fibers in recent years has further increased the revenue and profit of ramie-processing enterprises, presenting new opportunities for the ramie industry [7].

During long-term cultivation, ramie has undergone extensive artificial selection [8,9,10,11]. However, only within the past decade have high-throughput sequencing technologies enabled the generation of transcriptomic, genomic, and proteomic resources for this species [8,12,13,14]. These advances have facilitated the construction of genetic maps, the development of numerous molecular markers [15,16], the functional characterization of several genes [17,18], and the preliminary establishment of a secondary cell wall (SCW) synthesis model in ramie [14], as well as progress in understanding epigenetic and post-transcriptional regulation in ramie. In addition, efforts to establish a genetic transformation system have explored Agrobacterium-mediated transformation using explants or floral-dip methods, as well as particle bombardment, and successful transformation has been achieved in several ramie varieties [19,20,21,22].

Despite these advances, substantial gaps remain. Compared with cotton—the world’s most extensively studied fiber crop—ramie research began relatively late. As a result, functional validation of candidate genes remains limited; many GWAS/QTL signals lack mechanistic interpretation; the stability and efficiency of genetic transformation systems require further improvement; and a gene-editing platform has yet to be established. These limitations collectively slow the translation of molecular findings into practical breeding outcomes.

Given the specific industrial value of ramie and the increasing demand for high-quality ramie fibers, it is timely to summarize recent progress in this field. In this review, we follow the logical progression of molecular breeding to organize the current knowledge on ramie. We begin by outlining the breeding objectives for fiber quality and yield, and then summarize QTL and GWAS studies that help identify candidate genes and molecular markers aligned with these objectives. We further integrate recent advances in the molecular and cytological mechanisms of fiber development, together with progress in genomic resources that support the selection of appropriate reference genomes. Finally, we discuss the major molecular breeding approaches, including molecular markers, genomic selection, and genetic transformation, with particular attention to the current status and limitations of ramie transformation systems. By structuring the review in this way, we aim to provide breeders with a coherent reference that links breeding goals to the selection of target genes, markers, and genomic resources, and ultimately to the design of feasible molecular improvement strategies. It also highlights current knowledge gaps and future research directions that may ultimately accelerate the application of molecular breeding to improve fiber quality and yield in ramie.

## 2. Breeding Objectives

### 2.1. Reducing Colloidal Matter Content

Degumming is a critical step in ramie fiber production. Sun-dried ramie bast fibers contain a total colloidal matter content of 28.50% ± 1.29%, including 5.09% ± 0.24% pectin, 14.74% ± 0.54% hemicellulose, 0.95% ± 0.09% lignin, 1.27% ± 0.09% waxes, and 6.45% ± 0.03% water-soluble substances [23]. Microscopically, network- and block-like pectic structures are distributed on the membranous surface of hemicellulose between bast fiber cells, forming a pectin-derived membranous layer that fills the middle lamella. Because ramie bast fibers contain very low levels of lignin (Table 1), they are easier to degum than fibers such as jute or crop straws, whose middle lamellae contain high concentrations of lignin [24,25]. For context, hemp and flax also contain more lignin than ramie [1,23].

Incomplete degumming causes fibers to adhere to one another [26], which is undesirable for spinning. Ramie fabrics that are insufficiently degummed or inadequately treated often produce a pricking sensation during wear, primarily due to hairiness—cellulose-derived protruding fibers—on the yarn surface. Treatment with cellulase or pectinase removes this surface hairiness and softens the fibers, resulting in smoother fibers and a marked reduction in pricking [27,28,29]. Pectinase treatment produces a similar effect [27], likely because pectin removal softens the fibers, thereby reducing fiber breakage and the formation of hairiness [29]. However, traditional degumming processes remain costly and pose environmental concerns, whereas the more environmentally friendly biological degumming methods have not yet been fully adopted [25,30].

Among the water-insoluble colloidal components of ramie bast fibers, pectin accounts for 5.09%, second only to hemicellulose (14.74%) [23]. Pectin consists mainly of three classes: homogalacturonan (HG), rhamnogalacturonan-I (RG-I), and rhamnogalacturonan-II (RG-II). Ramie pectin is composed of approximately 63.3% HG and 36.7% RG-I [31]. The solubility of pectin depends on its degree of polymerization and the number and distribution of methoxyl groups [32]. However, unlike jute and other bast fiber crops, ramie pectin is difficult to remove effectively through natural retting [30,33,34], although it can be hydrolyzed by dilute acid or alkali solutions [13].

Hemicellulose is the major component of ramie bast fiber colloids and mainly includes galactoglucomannan, glucomannan, and xylan [23,26]. Compared with pectin, hemicellulose is more difficult to remove because its side chains crosslink with other molecules to form a network structure [13]. On the fiber surface, pectin-derived network and membranous structures cover the hemicellulose layer [25]. The removal of pectin exposes and partially removes the underlying hemicellulose, after which the hemicellulose membrane becomes exposed [35], creating conditions for its subsequent removal.

Recent studies have begun to identify genes that influence colloidal matter content in ramie fibers. GWAS analyses have detected multiple loci and candidate genes associated with pectin, hemicellulose, and other gum components, including 14 genes involved in polysaccharide synthesis and metabolism that may regulate hemicellulose deposition, and seven genes related to calcium transport or binding that may affect pectin composition [13]. In addition, QTL mapping combined with differential expression analysis has revealed three candidate genes potentially involved in lignin biosynthesis [36]. Notably, existing evidence suggests that pectin may be negatively associated with fiber growth. For example, the KNOX gene Bnt07G011994 may suppress fiber development by promoting pectin biosynthesis [17], while the pectin methylesterase gene Bnt14G019616 is positively associated with pectin content but negatively associated with fiber yield [18]. Both genes have been functionally validated through overexpression in *Arabidopsis*. These findings imply that reducing pectin content may not only lower degumming costs but also exert a positive effect on fiber growth.

Earlier literature has discussed the feasibility of reducing gum content in ramie through genetic modification [33], and with these recent advances, progress toward this breeding objective has become increasingly attainable.

**Table 1:** Mechanical and chemical properties of four natural bast fibers (hemp, flax, nettle, and ramie) [1,2,23,37,38,39].

Properties	Hemp	Flax	Jute	Nettle	Ramie
Elastic modulus (GPa)	45	70	-	87	130
Tensile strength (MPa)	1500	800	-	1600	500
Strain to failure (%)	0.8 (±0.1)	3.27 (±0.4)	-	0.9–2.5	2.5
Fineness (dtex)	25.547	3.0137	40.664	-	1.0998
Softness (twist/10 cm)	50.2	40	33.5	-	65.4
Tenacity (cN/dtex)	12	6.5	4.4	-	18
Elementary fiber length (mm)	15–25	17–25	1.5–5	20.9–28.7	20–250
Density (g/cm^3^)	1.35	1.38	-	1.45	1.5
Microfibril angle (deg)	6.2	10	8	-	3–7.5
Lignin (wt%)	3.7–5.7	2.2	12–13	-	0.95 ± 0.09
Hemicellulose (wt%)	17.9–22.4	18.6–20.6	13.6–20.4	-	14.74 ± 0.54
Pectin (wt%)	0.9	2.3	0.2	-	5.09 ± 0.24

### 2.2. Improving Fiber Quality and Yield

Ramie fibers possess significant mechanical advantages over other natural fibers. Microfibrils are helically wound within the cell wall, forming the structural framework of the fiber, and the angle of this winding strongly influences mechanical behavior. A smaller microfibril angle results in higher stiffness and more linear tensile elasticity, because tensile loading induces shear and hydrogen-bond breakage that leads to stick–slip behavior; once the microfibrils become fully aligned, the fiber exhibits elastic behavior again. Compared with hemp, flax, jute, and nettle, ramie exhibits superior rigidity, linear tensile elasticity, and dimensional stability due to its exceptionally low microfibril angle (3–7.5°) (Table 1), [1,40]. Moreover, ramie fibers are the finest, softest, and longest among bast fibers, Finer fibers allow a greater number of individual fibers to be incorporated into a yarn, which reduces slipping and increases strength. Therefore, tenacity is a particularly important property, as it reflects both fineness and strength. Ramie also has relatively long single fibers and can be spun in the form of individual fiber cells, giving it excellent spinnability, whereas some reports indicate that hemp fibers are difficult to separate into single fiber cells (Table 1), [2,41].

However, the high performance of ramie fibers does not preclude further improvement in quality and yield. In particular, the trade-offs among fineness, strength, and yield still require optimization through genetic improvement. Current studies on ramie fiber yield primarily focus on plant height, stem diameter, bark thickness, fiber yield percentage, stem number, and tiller number, whereas fiber quality is mainly evaluated based on fiber diameter and fineness.

**Figure 1:**
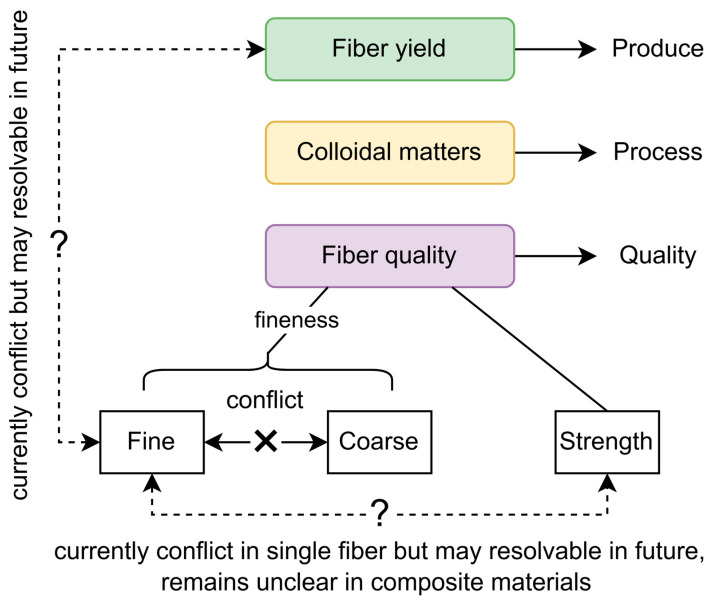
Conceptual framework illustrating the relationships between fiber yield, colloidal matter content, and fiber quality in ramie. Fineness can be improved toward either a “coarse” or “fine” breeding target. The “fine” direction currently shows a conflict with fiber yield based on existing studies, although this trade-off may become resolvable through future genetic improvement. Fineness also shows a conflict in single-fiber strength, and this trade-off may likewise be mitigated through genetic improvement, whereas its relationship in composite materials remains unclear.

It is important to note that “fiber fineness” does not literally refer to how thin a fiber is, but rather how long the fiber is per unit weight. Thus, fibers with higher fineness are not necessarily physically “finer”; they may instead have thinner cell walls [42]. Because cell walls are always deposited outside the plasma membrane, mature fiber cells do not increase in diameter but instead exhibit thicker secondary walls [43]. Across ramie varieties, yield-related traits such as plant height, stem diameter, bark thickness, and fiber diameter are negatively correlated with fiber fineness [44,45]. One possible explanation is that some ramie varieties accumulate more cellulose in their fiber cell walls [46,47], thereby increasing fiber yield. Nevertheless, it is not impossible to develop ramie varieties with both high yield and high fineness (Figure 1). While the number of primary fibers is relatively fixed within a species, the number of secondary fibers varies substantially [48]. Genes influencing fiber number [18], fiber fineness/diameter [46], and both traits simultaneously [47] have already been identified. Targeting such genes and combining them in more refined ways may enable the development of varieties that achieve both goals.

Although ramie indeed possesses the finest and softest fibers among bast crops and maintains high tensile strength [2], fibers with higher fineness tend to exhibit lower strength, which aligns with intuitive expectations [49]. As ramie fiber cells mature, they typically develop higher crystallinity and greater robustness in later stages [50]. This raises an important question: what is the purpose of pursuing higher fineness? If the goal is simply to obtain softer fibers, then not only low fineness but also high crystallinity—which contributes to strength—may conflict with the desired softness. Beyond textiles, studies in composite materials have shown that composite strength increases as fiber diameter decreases [51], but the mere incorporation of ramie fibers has also been shown to enhance composite strength [52]. However, comparative studies on the performance of ramie fibers with different fineness levels in composites are lacking. Considering that the innermost S3 layer of the fiber cell wall contains the highest cellulose content and exhibits the greatest strength [48], if increased fineness is achieved at the cost of reduced S3 deposition, the effect of fineness on composite strength remains difficult to predict (Figure 1).

This, in turn, raises another key question: does fiber strength arise from molecular-level structural organization within the cell wall, or simply from increased wall thickness? In *Arabidopsis* and other plants, the deposition patterns of the secondary cell wall are relatively well understood, and certain genes influence the molecular architecture of fibers by regulating the assembly of cellulose and lignin within the secondary wall [53]. No such regulatory mechanisms have yet been reported in ramie, but they may hold the key to enhancing fiber strength without altering fineness. For instance, fiber strength is strongly influenced by molecular-scale structural parameters: smaller microfibril angles and higher crystallinity generally confer greater tensile strength [54,55]. If these attributes can be modulated through molecular breeding, it may become possible to enhance fiber strength while maintaining desirable fiber morphology.

In summary, when defining breeding objectives, it is advisable to set two distinct targets for fiber fineness—one toward coarser fibers and one toward finer fibers (Figure 1). This approach not only provides greater flexibility for the textile industry but also accommodates the current uncertainty regarding how ramie fiber fineness influences the mechanical performance of composite materials. Fiber strength, meanwhile, should be treated as an independent breeding objective and clearly distinguished from fineness or fiber diameter.

## 3. Genetic Basis Underlying the Fiber Traits

### 3.1. Genetic Architecture of Fiber Quality Traits

Fiber quality encompasses length, strength, and fineness. While the length and strength of both cotton and ramie fibers are suitable for textile manufacturing, the fineness of ramie fiber is inadequate for high-quality fabric production, unlike that of cotton. Consequently, fineness is a critical determinant of ramie fiber quality and has been the focus of extensive research in previous studies. Before a reference genome for ramie was available, researchers sought to identify associations between molecular markers and fiber quality traits. Using this approach, the SSR marker RAM39 was found to be significantly associated with fiber fineness [56]. Expanding on this work, Chen et al. [57] increased the number of molecular markers and identified 52 SSR markers related to fiber fineness from a set of 1050. In recent years, advances in ramie genomics and its application to the genetic dissection of fiber traits have led to the identification of at least eight candidate genes associated with fiber fineness [45,58]. Among these, *Bng.009591*, an ethylene synthesis enzyme gene, harbors a SNP in its coding region that is significantly associated with fiber fineness [45]. Notably, fiber fineness declines rapidly during the late growth stage of ramie, a phenomenon that may be attributed to an increase in endogenous ethylene content. Through the construction of a segregating population derived from Zhongzhu No. 1 and Hejiangqingma, a high-density linkage map was developed, enabling genetic mapping of fiber fineness traits. This approach led to the detection of 58 quantitative trait loci (QTLs) for fiber fineness. Combined with dynamic transcriptome profiling and cytological observations, these findings further confirmed that ethylene-related genes regulate the formation of fiber fineness traits [44].

Ramie fiber is primarily composed of lignin, cellulose, hemicellulose, and pectin, and the content of these components significantly influences fiber quality. Association analysis revealed that a variant in the cellulose synthase gene *Bng.008687* is significantly associated with fiber fineness [45]. Meanwhile, a genome-wide association study (GWAS) of fiber gum components identified a total of 14 candidate genes related to sugar metabolism, which may be associated with hemicellulose and other components of the fiber gum [13]. Furthermore, genetic mapping using an F_2_ segregating population detected five QTLs for fiber lignin content. Combined with expression analysis, candidate genes were identified for three of these QTLs. Among them, the candidate gene for *qLC7* is an *MYB* transcription factor gene, which harbors numerous insertions and deletions in its coding sequence [36]. This indicates that variations in regulatory genes related to lignin biosynthesis have a significant impact on fiber quality.

### 3.2. Genetic Architecture of Fiber Yield and Its Related Traits

#### 3.2.1. QTL Analysis for Fiber and Its Related Traits

Ramie fiber is extracted from the stem bark, and consequently, fiber yield is a composite trait determined by component traits including ramet number, stem length, stem diameter, bark thickness, fresh bark weight, and fiber output ratio. A common approach for gene identification involves constructing linkage maps using segregating populations and performing subsequent trait linkage mapping (Table 2). The first genetic linkage map of ramie consisted of 132 SSR markers and spanned 2265.1 cM [59]. Using this map, a total of 33 QTLs were identified, 24 of which showed overdominance effects, suggesting strong heterosis for traits in ramie. Additionally, with Qingdaye and Zhongzhu 1 as parents, a high-density genetic map comprising 4338 molecular markers was constructed using the genotyping-by-sequencing technique, covering 1942.9 cM [60]. The following genetic analysis revealed four QTLs for fiber yield, and two to six QTLs for each of the fiber yield-related traits [60,61]. Among these, *qBT4a* is a QTL for bark thickness, and its candidate gene is predicted to encode an MYB protein. In the Zhongzhu 1 parent, this gene carries a 760-bp insertion within an exon, leading to premature termination of protein synthesis [60]. Similarly, *qSL11b* is a QTL for stem length, and this interval contains three DELLA protein genes. Given that DELLA proteins are key repressors of gibberellin signaling and inhibit plant growth, they are considered strong candidate genes for this QTL [61]. Importantly, QTL analysis for traits related to fiber yield using an F_1_ segregating population detected numerous QTLs, among which 23 were identified as pleiotropic loci that jointly control fiber yield and fiber fineness, thus providing a genetic explanation for the negative correlation between these two traits [44].

The wild ramie species *B. nivea* var. *tenacissima* shows low fiber yield, suggesting that numerous fiber yield-related genes underwent selection during domestication [10]. An F_2_ segregating population was developed using *B. nivea* var. *tenacissima* and the cultivated variety Zhongsizhu 1 as parents. From this population, a genetic map comprising 6433 markers and spanning 2476.5 cM was constructed [62]. Genetic mapping identified a total of 47 QTLs associated with fiber yield. Notably, only 14 of these QTLs carried beneficial alleles from the wild parent, providing further evidence that most fiber yield QTLs were subject to selection during domestication. For instance, *qFY5*, a major QTL for fiber yield, was identified, and the auxin-responsive gene *Bnt05G007931* was proposed as its candidate gene based on expression analysis. The genomic region harboring this gene exhibits pronounced differentiation between wild and cultivated ramie, with significantly reduced nucleotide diversity in the cultivated variety. These findings confirm that this QTL underwent domestication selection [10]. Additionally, a major QTL for leaf fiber content was also detected in this population. Its candidate gene, *whole_GLEAN_10016511*, encodes an MYB transcription factor, and two large insertion/deletion fragments were identified in its promoter region between the two parents [63]. Overexpression of this candidate gene in *Arabidopsis* resulted in a significant increase in lignified fiber bundles in the transgenic plants, indicating that this gene can regulate fiber formation and may contribute to ramie fiber yield.

#### 3.2.2. GWAS for Fiber Yield Traits

The rich genetic diversity of ramie germplasm resources thereby positions association analysis using natural populations as a powerful approach to dissect the genetic architecture of its yield-related traits (Table 3). Luan et al. [64] identified 16 SSR markers associated with fiber yield traits in a panel of 107 ramie core accessions. Specifically, marker RAM290 was significantly associated with stem diameter across three environments, explaining between 8.32% and 23.42% of the phenotypic variation [64]. Nevertheless, as this initial study used only 95 SSRs for association analysis, its findings warranted further validation. The application of specific-locus amplified fragment sequencing (SLAF-seq), a high-throughput marker identification technique, enabled a more comprehensive analysis. Using this approach, 215,376 SNP markers were detected in 112 core accessions, among which 42 were found to be significantly associated with fiber yield-related traits [58], and an additional 44 were associated with ramet number [65].

The release of the ramie genome sequence has enabled the construction of whole-genome variation maps via germplasm resequencing. Resequencing 319 core accessions identified 5.78 million variants, which were then used to analyze linkage disequilibrium (LD), revealing an average genome-wide LD decay distance of 30.28 kb. A subsequent genome-wide association study (GWAS) on four yield-related traits—plant height (PL), stem diameter (SD), bark thickness (BT), and ramet number (RN)—identified 265 associated loci, 60 of which (containing 977 genes) were pleiotropic [45]. Haplotype and expression analyses further suggested that *BnNAC29* may play a dual role in regulating both stem diameter and bark thickness. Additionally, Zeng et al. [18] resequenced 301 ramie accessions, generating 6.73 million markers and achieving a genome-wide marker density of 24.9 markers per kb. A subsequent association analysis for six fiber yield-related traits detected a total of 129 significant signals [18]. Among these, *Bnt03G004997*, an NAC protein-encoding gene, harbored promoter variants significantly associated with the fiber output ratio. Moreover, variants in the coding region of *Bnt10G015830* were significantly associated with bark weight and fiber yield traits. This gene contains a single-base deletion in its exon, which causes a frameshift in the encoded protein. The ramie germplasm ‘1380’ fails to produce fibers in its stem bark. Using this germplasm as a parent, a segregating population was developed, leading to the detection of a major QTL on chromosome 14. Through the use of recombinant lines, this locus was subsequently fine-mapped to a 168 kb region. Notably, several variations within this region was found to be significantly associated with both fiber yield and bark weight traits. This associated locus resides in the gene *Bnt14G019616*, which encodes a pectin methylesterase. The pectin content in the stem bark of ‘1380’ was significantly higher than that in other varieties, further supporting the critical role of pectin deposition and metabolism in fiber formation [18].

### 3.3. QTL, GWAS Analysis for Other Bast Fiber Crops

Similar to ramie, other bast fiber crops such as flax, hemp, jute, and kenaf have also seen substantial progress in QTL mapping and association studies (Table 4), [66,67]. Except for dioecious hemp, RIL populations have been developed in these species for QTL identification. As in ramie, fiber-yield-related traits have received considerable attention. Notably, in flax, an SSR-based association study using the MLM model identified a gene associated with fiber strength [68]. In jute, six epistatic QTLs related to fiber strength were detected, and phenotypic analyses indicated that fiber fineness and fiber strength behave as largely independent traits [69].

In ramie, fiber strength has rarely been included in QTL or association analyses, despite being an important component of fiber quality. Therefore, findings from flax and jute provide valuable reference points for future research. In addition, cellulose content is another key determinant of bast fiber quality; in jute, five QTLs associated with cellulose content in bast fibers have been identified [70]. In kenaf, a QTL associated with elution rate was detected, potentially contributing to breeding efforts aimed at reducing gum content [71].

**Table 4:** Summary of QTL and association studies related to bast fiber traits in other bast fiber crops.

Crop	Study Type	Trait(s)	Population Size	Ref.
flax	SSR association	plant height, number of branches	390	[72]
flax	SSR association	fiber yield, fiber length, fiber strength	264	[68]
flax	QTL mapping	fiber components, straw weight, height	(RILs) 243	[73]
flax	QTL mapping	plant height, technical length	(F7) 110 + (R7) 123	[74]
flax	QTL mapping	plant height, stem length, stem yield, fiber yield, fiber content	(F2) 122	[75]
flax	GWAS	plant height, technical length, number of branches	224	[76]
flax	GWAS	plant height, technical length, fiber content	224	[77]
flax	GWAS	plant height	260	[78]
flax	GWAS	plant heigh	220	[79]
flax	GWAS	plant height, technical length, technical weight, number of nodes, stem diameter, fiber content, fiber weight, fiber length, distance between internodes, stem slenderness index, stem tapering index	306	[80]
hemp	GWAS	cell walls, fiber content	123	[81]
jute	QTL mapping	fiber yield, fiber fineness, fiber strength	(F6) 120	[69]
jute	QTL mapping	fiber fineness, fiber yield, number of nodes, plant height, stem diameter, tensile strength	(F6) 120	[82]
jute	QTL mapping	fiber yield, plant height, stem diameter	(F2:3) 176	[83]
jute	QTL mapping	cellulose content of bast fiber	(F2) 104	[70]
kenaf	QTL mapping	plant height, stem diameter, bark thickness, stem weight, bark weight, fiber weight, elution rate	(F7:8) 138	[84]
kenaf	QTL mapping	plant height, stem diameter, skin thickness, stem weight, bark weight, cellulose content	(F2:3) 146	[71]
kenaf	GWAS	plant height, dry weight	96	[85]

## 4. Molecular Mechanisms of Fiber Development

### 4.1. Patterned Deposition of the Secondary Cell Wall and the NAC–MYB Network

Deposition of the plant secondary cell wall (SCW) is controlled by a highly conserved NAC–MYB transcriptional regulatory network. Secondary wall NAC master switches (SWNs) occupy the top tier of this hierarchy and activate cascades of MYB transcription factors involved in the biosynthesis of cellulose, hemicellulose, and lignin, thereby initiating the full secondary wall formation program [53].

This regulatory hierarchy is broadly conserved in ramie, and a secondary-wall formation model has now been established for this species [14]. Auxin functions as an upstream signal that initiates SCW biosynthesis, partly through the vacuolar auxin transporter WAT1. In ramie, the WAT1-encoding gene *CL12943Contig1* shows signatures of positive selection during domestication and is associated with bark thickness [62]; similarly, another WAT1 homolog, *Whole_GLEAN_10007759*, is associated with fiber percentage [58]. In addition, several auxin-responsive genes, such as *whole_GLEAN_10025406* and *Bnt05G007931*, are linked to fiber-yield traits [10,58]. These findings suggest that auxin-mediated activation of SCW formation may influence fiber yield and thus represents a plausible breeding target.

NAC transcription factors act as first-layer master switches [86]. In ramie, NAC genes are frequently associated with multiple yield-related traits [45,58,62]. Overexpression of ramie NAC homologs—including *BnNAC29*, *Bnt05G007257* (*SND2*), *Bnt03G004997* (*VND4/5*), and *Bnt08G012573* (*NST1/2*)—in *Arabidopsis* increases fiber cell number [10,18,45,47], supporting their conserved regulatory roles. The second-layer master switches, *MYB46* and *MYB83*, coordinate downstream transcription factors to regulate SCW thickening [86]. In ramie, several MYB genes have been associated with fiber yield and fineness, such as *evm.model.scaffold7373.133_D1*, *Bng.000585*, *Bng.076290*, and *Bng.064534* [45,60]. Functional validation in *Arabidopsis* has also been achieved for multiple ramie MYB genes, including *whole_GLEAN_10015497*, *BntWG10016451*, and *whole_GLEAN_10016511*, all of which increase fiber cell number upon overexpression [63,87,88]. Notably, *whole_GLEAN_10016511* is associated with crude fiber content in ramie leaves and is differentially expressed among varieties with contrasting crude-fiber levels; its overexpression enhances stem fiber content in *Arabidopsis*, suggesting a broader functional spectrum. Among downstream transcription factors, *KNAT7* is a direct target of *MYB46* [89]. Overexpression of its ramie homolog *Bnt07G011994* suppresses xylem development in *Arabidopsis* [17], and overexpression of the KNAT1 homolog *whole_GLEAN_10029667* produces a similar reduction in fiber formation [90].

Among downstream structural genes, PRX is included in the lignin-biosynthesis module of the ramie SCW model [14], and the PRX gene *Bnt10G015742* is associated with higher bark and fiber yield [18]. RWA3, involved in hemicellulose biosynthesis, also supports the applicability of the SCW model to ramie: overexpression of its ramie homolog *whole_GLEAN_10024150* in *Arabidopsis* increases xylem fiber number and wall thickness [87].

These findings indicate that ramie possesses a conserved secondary-wall formation program comprising transcriptional regulators, structural genes, and cytoskeletal components. In addition, the *APL*–*NAC86* cascade has been shown to regulate phloem fiber differentiation, while ethylene and gibberellin signaling synergistically promote secondary-wall thickening, suggesting extensive crosstalk between hormonal pathways and transcriptional regulators during bast fiber development [43,44]. Therefore, hormone-related genes may also influence fiber traits in ramie. For example, overexpression of the ACC oxidase gene *Marker00009591* increases fiber fineness in ramie [46]. In addition, the auxin-responsive genes *Bnt05G007931* and *whole_GLEAN_10025406* have been associated with fiber-yield traits [10,58].

In *Arabidopsis*, secondary wall cellulose biosynthesis primarily involves the CesA4/7/8 cellulose synthase complex, and the microtubule cytoskeleton determines the spatial pattern of deposition by guiding the movement of cellulose synthase complexes [53,91,92,93]. Several *CesA* genes have been identified in ramie, and at least one of them shows structural variation between cultivated and wild accessions [8,10], which may contribute to the distinctive properties of ramie fibers. Microtubule-associated proteins also influence SCW deposition. For example, *Bng.042170* (kinesin-like protein KIN-7D) and *Bng.086916* (KINESIN LIGHT CHAIN-RELATED 1) are associated with fiber fineness [45], consistent with the role of the cytoskeleton in guiding cellulose synthase trajectories during patterned SCW deposition.

Notably, the concept of the tertiary cell wall (TCW) has emerged in recent years to describe an inner, highly crystalline, low-lignin wall layer in fiber crops, distinct from the primary cell wall (PCW) and SCW [48]. The cited review [48] proposed that ramie may also possess a TCW; however, no ultrastructural or omics-level evidence has yet been reported to confirm its presence. Further validation is needed. If TCW formation in ramie is eventually demonstrated, it may provide new insights into the exceptionally high crystallinity and low lignin content characteristic of ramie fibers.

To date, a subset of fiber-related genes in ramie has been functionally validated, and these genes may serve as potential targets for molecular breeding (Table 5).

### 4.2. Epigenetic and Post-Transcriptional Regulation

Non-coding RNAs (ncRNAs) play essential regulatory roles in plant growth, development, and stress responses [94]. In ramie, numerous regulatory ncRNAs have been identified, with microRNAs (miRNAs) being the most extensively studied class [95]. These miRNAs silence target mRNAs through sequence complementarity and exhibit tissue- and stage-specific expression patterns, including differential expression in bast fiber cells [96,97,98]. In addition to miRNAs, long non-coding RNAs (lncRNAs) and circular RNAs (circRNAs) have also been implicated in fiber development [88,99]. For example, lncR00022274 may suppress fiber formation by interacting with the gene *BntWG10016451* [88], suggesting that ncRNAs could serve as promising targets for improving fiber traits.

At the protein level, post-translational regulation provides an additional layer of control over fiber development. Proteomic analyses in ramie have revealed diverse structural and metabolic proteins associated with fiber formation [14]. Many of these proteins undergo post-translational modifications, such as ubiquitination and phosphorylation. For instance, MYB transcription factors and RWA3 homologs are regulated through ubiquitin-dependent protein degradation pathways [87]. Phosphorylation also plays a critical role in fiber growth, as demonstrated by the dynamic kinase- and phosphatase-mediated regulation of KNOX proteins, which act as inhibitors of fiber development [90]. These findings highlight the importance of protein-level regulation in linking gene expression to fiber phenotypes.

## 5. Ramie Genome and Its Evolution

With ongoing improvements in sequencing technology, the genomes of at least four ramie varieties have been sequenced and assembled since 2018, resulting in six versions. The increasing quality of these assemblies has, in turn, driven advances in ramie genetics and breeding.

### 5.1. Genome Assembly and Annotation

#### 5.1.1. Ramie Assemblies from Illumina Sequencing

The first draft genome of ramie was released in 2018. The research team sequenced the Zhongzhu 1 genome on the Illumina HiSeq 2500 platform at approximately 85-fold coverage, using four constructed libraries with insert sizes of 230 bp, 500 bp, 2 kb, and 5 kb. The final assembly, totaling 335.6 Mb with a scaffold N50 of 42,283 bp, covered an estimated 74.9% of the genome (estimated size 448 Mb). It contained 58.89% repetitive sequences and exhibited a heterozygosity rate of approximately 1.48%, with CEGMA evaluation confirming the successful assembly of 97.6% of core eukaryotic genes [8]. A second ramie draft genome was released later that year, achieved with 256-fold sequencing coverage. The assembly comprised 341.9 Mb, with a scaffold N50 of 1126.36 kb. Repetitive sequences made up 46.3%, and the heterozygosity rate was 3.1%. BUSCO analysis of 1440 eukaryotic genes showed 90.5% completeness, including 84% single-copy genes [95]. However, the two ramie genome assemblies generated via next-generation sequencing both exhibit severe fragmentation.

#### 5.1.2. Ramie Assemblies from PacBio Sequencing

The application of long-read sequencing technologies has markedly improved the continuity of ramie genome assemblies. By integrating Nanopore, PacBio, Hi-C, and Illumina sequencing, complete de novo genome assemblies were generated for the wild species *B. nivea* var. *tenacissima* and the cultivated variety ‘Zhongsizhu 1’, resulting in genome sizes of 270.2 Mb and 266.6 Mb, respectively (Table 6) [10]. The scaffold N50 values reached 19.55 Mb and 17.80 Mb, with 97% of the wild and 93% of the cultivated sequences anchored to 14 chromosomes. The assemblies achieved a BUSCO completeness of 96.9%. LTR Assembly Index (LAI) scores classified the wild genome as gold (23.38) and the cultivated genome as reference level (19.28), marking them as the most complete ramie genomes to date [10]. The ‘Zhongzhu No. 1’ genome was assembled in 2023 using a combination of PacBio RS II and Illumina sequencing, producing a 241.85 Mb assembly with a scaffold N50 of ~17.61 Mb and 90.2% of sequences anchored to 14 chromosomes. BUSCO analysis indicated 98.1% completeness, and the LTR Assembly Index (LAI) was 14.47 [44]. Using PacBio Sequel II long-read sequencing, the genome of HZS10—a feral ramie individual collected from the field—was assembled into 294 Mb, achieving a scaffold N50 of 17.61 Mb and a contig N50 of 3.42 Mb. The assembly comprised 54.85% repetitive sequences and demonstrated a BUSCO completeness of 95.8% (Table 6) [100]. Notwithstanding the enhanced continuity afforded by long-read sequencing, extant ramie genome assemblies contain hundreds of contigs, indicating that further refinement is required (Table 6). To obtain a more comprehensive genomic resource, a telomere-to-telomere (T2T) complete genome has been assembled for the landrace ‘1380’, totaling 344 Mb and consisting of only 14 contigs, thereby reflecting gapless continuity across all chromosomes.

#### 5.1.3. Genome Annotation

Predicted gene numbers vary considerably across different ramie genome assemblies. The two initial Illumina-based assemblies annotated 42,463 and 30,237 genes, respectively [8,95]. In contrast, genome assemblies based on PacBio sequencing generally yielded fewer predicted genes than these Illumina-based versions, including 20,693 for the wild B. nivea var. tenacissima, 19,498 for ‘Zhongsizhu 1’, 27,664 for the ‘Zhongzhu 1’ assembly by Chen et al. [44], and 22,312 for HZS10 (Table 6). This discrepancy may be due to redundancy resulting from the fragmentation of Illumina-based assemblies (Table 6). In the telomere-to-telomere (T2T) complete genome of ‘1380’, 25,853 protein-coding genes and 3325 non-coding RNAs were annotated. Furthermore, annotations across these genome versions indicate that repetitive sequences account for approximately 46% of the ramie genome. For instance, transposable elements constituted 46.3% of the Zhongzhu 1 genome, including 8.2% DNA transposons and 33.8% LTR retrotransposons [8]. Similarly, in the wild *B. nivea* var. *tenacissima* and Zhongsizhu 1, repetitive sequences comprised 112.7 Mb (45.5%) and 117.1 Mb (44.3%) of the genomes, respectively, with LTR retrotransposons accounting for 40.5% and 39.1%. However, in the more recent HZS10 and T2T assemblies, repetitive sequences accounted for 54.85% and 61.19% of the genomes, respectively—markedly higher than in earlier versions [100]. This indicates that a substantial portion of repeats remained unassembled in previous versions due to limitations in assembly quality.

### 5.2. Genome Evolution

Phylogenetic analysis revealed that ramie shares the closest evolutionary relationship with white mulberry (*Morus alba*), from which it diverged approximately 48.7 million years ago [10]. Comparative analyses of gene families across ten species revealed 1075 gene families unique to ramie, encompassing 4082 genes. Notably, these included five putative cellulose synthase genes, suggesting a potential link to the unique fiber properties of ramie [8]. A further comparative analysis of 14 species revealed that, among the 14,295 gene families identified in ramie, 706 were unique. Extending this comparison to four species—*Arabidopsis thaliana*, *M. notabilis*, ramie, and jute (*Corchorus* sp.)—identified 1118 ramie-specific gene families. This finding reinforces the notion of ramie’s genetic uniqueness within the Rosales order and among other fiber crops [95]. To investigate whether evolutionary changes in the ramie genome contributed to fiber formation, Wang et al. [10] analyzed the expansion of 85 known fiber development-related gene families across several fiber crops. The results demonstrate that kenaf, flax, hemp, and cotton expanded 56, 57, 18, and 10 of these families, respectively, while only four and three showed expansion in ramie and jute. This limited expansion in ramie may result from a single ancient hexaploidization event with no recent whole-genome duplications (WGDs), and therefore suggests that its distinctive fiber traits may stem from an evolutionary pathway distinct from that of other fiber crops.

Investigation of chromosomal evolution revealed that ramie and mulberry have retained 14 chromosomes, whereas jujube and fig—also belonging to the Rosales order—possess only 12 and 13 chromosomes, respectively [10]. This difference suggests that chromosomal breakage and fusion occurred during ramie evolution. Synteny analysis traced chromosomal evolution from jujube, an early Rosales species, to the common ancestor of ramie, mulberry, and fig. During this transition, nine chromosomes remained largely intact, while the other three underwent splitting and fusion, generating five new chromosomes. Ramie and mulberry have fully retained these 14 chromosomes. In fig, however, two of these chromosomes subsequently merged, reducing the total to 13.

### 5.3. Domestication, Improvement and Feralization

The wild subspecies *B. nivea* var. *tenacissima* is widely accepted as the progenitor of cultivated ramie. As is common in crop evolution, the development of cultivated ramie encompassed both domestication and subsequent breeding improvement. More recently, however, the contraction of the ramie industry has led to the abandonment of many varieties, and consequently, their descendants display a distinct feralization trend.

#### 5.3.1. Ramie Domestication

Wild and cultivated ramie exhibit extensive genetic divergence. A comparison of their genomes identified 13,090 shared gene families, along with 1276 and 1068 specific families in the wild and cultivated varieties, respectively. Functional enrichment analysis revealed that wild-specific genes were primarily enriched in RNA polymerase-related categories, whereas cultivated-specific genes were enriched in plant hormone signal transduction and photosynthesis pathways [10]. Comparative genomic analysis of the cultivated variety ‘Zhongzhu 1’ and four wild species (*B. nivea* var. *nipononivea*, *B. nivea* var. *viridula*, *B. nivea* var. *tenacissima*, and *B. nivea* var. *nivea*) confirmed multiple gene flow events among these taxa and identified 269 positively selected genes that were also enriched in pathways related to photosynthesis and hormone response. These results indicate that genes governing growth regulation and photosynthetic efficiency were subject to directional selection during domestication [11].

Both wild and cultivated genomes harbor abundant structural variations (SVs), including 5687 insertions/deletions, 3700 copy number variations, 1880 inversions, and 1162 translocations. The largest SV, located on chromosome 4, encompasses 38 genes, among which *Bnt04G005505*, *Bnt04G05508*, and *Bnt04G005520* exhibited differential expression in developing stem bark. Furthermore, an inversion and translocation in adjacent regions on chromosome 1 of the cultivated genome resulted in the duplication of a *CesA* gene, potentially influencing fiber synthesis [10]. Despite the lack of clear genome-wide differentiation between wild and cultivated ramie populations (FST = 0.011), nucleotide diversity analysis revealed 186 domestication regions spanning 7.36% of the assembled genome. Within these selective sweeps reside 92 fiber development-related genes, 11 of which are known transcriptional regulators. For instance, *Bnt08G012573*, encoding an NAC transcription factor orthologous to *Arabidopsis* NST1/NST2, significantly increased phloem fiber number upon overexpression in *Arabidopsis*. Notably, both the fiber yield QTL *qFY5* and the fiber elongation-associated gibberellin metabolism gene *BntGA2ox1* were identified as targets of domestication selection, implicating their roles in the evolution of fiber traits during ramie domestication.

#### 5.3.2. Improvement of Fiber Traits in Ramie Breeding

Cultivated ramie varieties exhibit extensive genetic diversity in agronomic performance. Zeng et al. [18] resequenced 254 landraces and 47 improved cultivars, constructing a genome-wide variation map of ramie germplasm. Nucleotide diversity analysis revealed that, compared to landraces, improved cultivars showed significantly reduced diversity in 173 genomic regions, spanning 17.5 Mb and covering approximately 6.48% of the genome. These selective sweeps were concentrated on chromosome 9, which accounted for 40.5% of all selected regions, indicating that nearly 40% of this chromosome experienced intensive selection during breeding. By integrating transcriptomic and proteomic data across fiber developmental stages, 156 fiber development-related genes were identified as targets of breeding selection. Among them are the *NAC* gene *Bnt05G007257* and four *MYB* genes (*Bnt04G006700*, *Bnt07G011845*, *Bnt09G014274*, and *Bnt09G014294*), all closely related to *Arabidopsis* NAC and MYB regulators of secondary wall synthesis. Interestingly, *Bnt07G011845* exhibits six haplotypes, with only two retained in improved cultivars—a pattern indicative of strong selection during breeding. Furthermore, four gibberellin-related genes were identified as targets of selection. These comprised two *GA2ox* genes (*Bnt13G019022* and *Bnt13G019023*) involved in gibberellin biosynthesis, a *GID1C* gene (*Bnt02G003447*) encoding a gibberellin receptor, and a *DELLA* gene (*Bnt09G013717*) functioning in gibberellin signaling. The Hap4 haplotype of *Bnt09G013717* is specific to improved cultivars and may be associated with breeding selection for plant height.

#### 5.3.3. Ramie Feralization

Over the past few decades, the continuous decline in ramie cultivation, together with the easy dispersal of its small seeds and subsequent feral growth, has driven the widespread feralization of many cultivated varieties. A genomic investigation into the mechanisms underlying this feralization revealed that feral populations are genetically positioned between wild and cultivated ramie, yet they are more closely related to the cultivated types. Feral ramie exhibited the highest level of genetic diversity, in marked contrast to the lowest diversity observed in wild populations. Inference using the DIYABC Random Forest algorithm indicated that feral ramie originated from hybridization between wild and cultivated types, with 90.7% of its specific SNPs attributable to cultivated varieties and 9.3% to wild populations. This genomic constitution provides a compelling explanation for the closer ecological similarity of feral ramie to cultivated types. A total of 728 selected regions were identified in feral ramie, in contrast to the 605 found in domesticated ramie. These regions exhibited enrichment in distinct stress-response pathways, suggesting that feral and domesticated ramie have been molded by different environmental pressures. Additionally, feral ramie developed specific adaptations to temperature, precipitation, and soil nutrient availability [100].

## 6. Molecular Breeding Tools and Technical Systems

### 6.1. Molecular Markers

Molecular markers have been widely applied in germplasm evaluation and trait analysis. With technological advances, their primary applications have gradually shifted from early rapid screening to high-resolution genomic analysis. SRAP, ISSR, and RAPD are fingerprint-type molecular markers that do not require prior sequence information. These methods are low-cost, simple to perform, and suitable for rapid large-scale screening, and are therefore commonly used in early-stage studies or for preliminary assessments [15,101,102,103,104]. For example, Liu et al. [15] used 33 pairs of SRAP primers to analyze the genetic relationships between 35 ramie genotypes from Brazil and China; Wang et al. [102] used seven ISSR primers to assign 16-bit binary molecular IDs to 42 ramie accessions. It is important to note that fingerprint markers are highly sensitive to PCR conditions and exhibit poor reproducibility and cross-laboratory comparability. For instance, RAPD produces different banding patterns among different clones of the same ramie variety [101]. Therefore, before interpreting such differences as population-level variation, technical replicates or validation using higher-resolution markers are recommended.

SSR and EST-SSR markers, which offer higher reproducibility and can distinguish heterozygous from homozygous genotypes, have long been used for germplasm evaluation, core collection construction, and low-density linkage map development. Because repeat regions are prone to variation during replication, repair, and recombination, while flanking regions remain relatively conserved, primers designed to amplify these regions generate polymorphic bands after electrophoresis. SSRs derived from transcriptome sequences are known as EST-SSRs. Thousands of EST-SSR markers have been developed in ramie, and their polymorphisms have been validated for germplasm identification [12,57,105,106]. Genome-derived SSRs were later reported [16,107] and have supported germplasm evaluation, core collection development, and molecular ID systems in subsequent studies.

Although ramie possesses a considerable number of SSR loci, constructing high-density genetic maps using SSRs is relatively costly. With declining sequencing costs, genome-wide SNPs have become the predominant marker type, offering much higher resolution for high-density genetic mapping, GWAS, and genomic selection [8,65]. The availability of ramie genome assemblies [8,44,95] has enabled large-scale SNP discovery, laying the foundation for subsequent locus mapping and population genomics research [8,44,65].

In summary, fingerprint markers such as RAPD, ISSR, and SRAP do not require prior sequence information and allow rapid, low-cost screening, but their poor reproducibility limits downstream applications. SSR markers provide higher accuracy and cross-laboratory consistency and are suitable for germplasm identification and low-density linkage mapping, although their resolution is lower than that of SNP-based markers. SNPs, which occur in extremely high abundance across the genome, enable the construction of high-density genetic maps. As discussed above, these SNP-based maps have facilitated the identification of numerous QTLs and QTNs associated with fiber-related traits, providing valuable resources for marker-assisted selection in ramie breeding.

### 6.2. Genetic Transformation Systems

#### 6.2.1. Transgenic Methods

**Figure 2:**
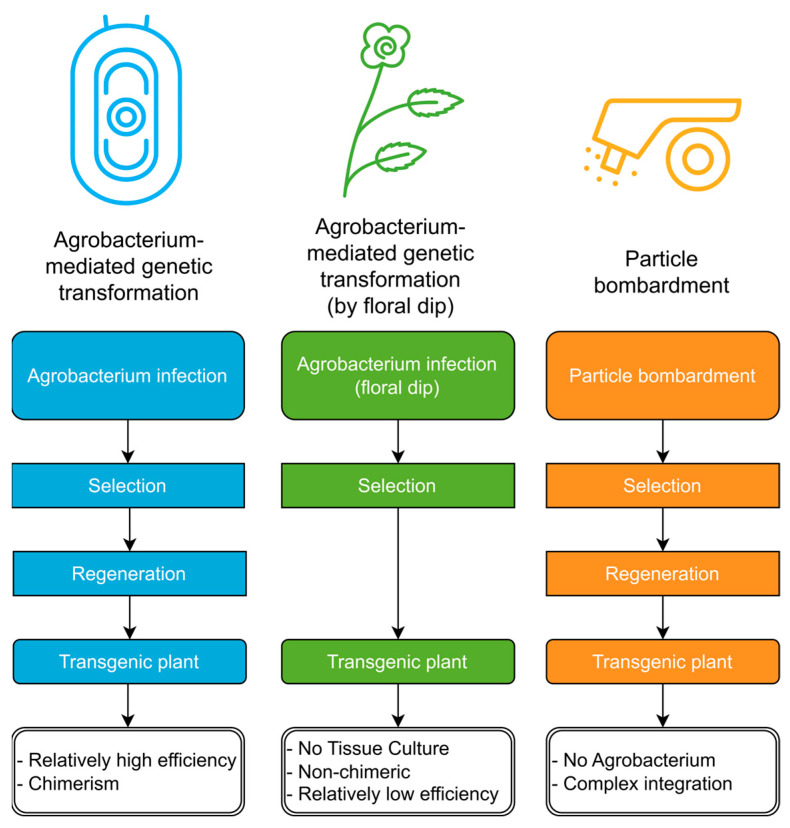
Three existing genetic transformation methods in ramie. Agrobacterium-mediated transformation using leaf or seedling explants generally achieves relatively high efficiency but often results in chimeric plants. Agrobacterium-mediated floral dip does not require tissue culture and can produce non-chimeric progeny, although its efficiency is low. Particle bombardment is independent of Agrobacterium but frequently leads to complex integration patterns.

Three genetic transformation methods have been established in ramie: Agrobacterium-mediated transformation using leaf or seedling explants, Agrobacterium-mediated floral dip, and particle bombardment. These methods differ in workflow, efficiency, and transgene integration patterns (Figure 2).

Agrobacterium-mediated transformation remains the most widely used approach in ramie. Since the first successful report in 1993 [108,109], various explant types have been tested, including sexually derived cotyledons and hypocotyls [22,110,111,112,113,114,115,116,117], as well as vegetatively derived leaf discs, leaf midribs, petioles, stem segments, and callus tissues for Agrobacterium-mediated transformation [19,20,21,108,109,115,118,119]. All of these approaches ultimately rely on callus induction followed by regeneration of new plantlets. However, the efficiency and stability of shoot regeneration from transformed calli remain suboptimal, likely reflecting differences in regeneration capacity among ramie varieties, which will be discussed later.

In addition, an Agrobacterium-mediated floral dip method analogous to that used in *Arabidopsis* has also shown relatively high transformation efficiency, yielding approximately one transgenic seed per 115 treated seeds on average [22]. However, some studies reported failure to obtain transgenic seeds [120]. This discrepancy may stem from methodological differences: the former study immersed inflorescences in *Agrobacterium* suspension for several consecutive days, whereas the latter applied only a single spraying treatment. For this reason, immersion over multiple days is recommended when performing floral dip-like *Agrobacterium* inoculation in ramie. This method bypasses the need for regeneration and avoids chimerism, but it requires flowering plants and inevitably leads to segregation in the progeny.

Current reports on ramie Agrobacterium-mediated transformation, including both explant-based and floral dip methods, exclusively use *Agrobacterium tumefaciens* strains such as LBA4404, EHA101, and EHA105 (Table 7); no studies have reported the use of *Agrobacterium rhizogenes*.

Beyond *Agrobacterium*, only one study has reported particle bombardment in ramie, and its transformation efficiency was lower than that of Agrobacterium-mediated methods [21,22]. A likely explanation is that the study delivered whole plasmids, which tend to produce complex integration patterns, including frequent transgene rearrangements, multiple copies, and incorporation of vector backbone sequences. The use of linear minimal expression cassettes may help mitigate these issues [121]. A notable advantage of particle bombardment is that it does not require *Agrobacterium* suppression antibiotics, thereby reducing negative effects on regeneration. Although particle bombardment offers certain unique advantages, the available protocol remains preliminary, and additional optimization will be necessary to enhance its efficiency and reliability.

#### 6.2.2. Segregation Issues in Sexually Derived Transgenic Receptors

Ramie is highly heterozygous, which leads to pronounced trait segregation in progeny [19,59]. Consequently, when sexually derived materials are used as transformation receptors or for phenotypic evaluation, it becomes difficult to determine whether an observed phenotype is caused by the introduced gene or by segregation of the progeny’s genetic background. Therefore, using sexually derived explants introduces two layers of randomness when attempting to obtain transgenic ramie plants suitable for production:

(1) The probability that segregating progeny will exhibit desirable traits;
(2) The probability that random transgene insertion will not negatively affect gene expression or phenotype.

The overall likelihood of obtaining usable transgenic seedlings is approximately the product of these two probabilities, and both sources of randomness complicate the evaluation of transgene effects. Considering these factors, vegetatively propagated materials are recommended as preferred transformation receptors (Table 7).

#### 6.2.3. Regeneration of Transgenic Plants

Ramie varieties differ dramatically in their regeneration capacity. For example, the cultivar “Xiangsi Ramie C” exhibits a regeneration frequency of 96.67%, whereas “Dazhu Huangbaima” regenerates at only 0.1% [122,123]. Although differences in gene expression associated with regeneration ability have been explored, no major-effect gene controlling this trait has yet been identified [123]. Therefore, before conducting transformation experiments, it is advisable to evaluate the regeneration capacity of the chosen variety or select varieties with documented regeneration success (Table 7).

Current regeneration protocols for transgenic ramie can be broadly categorized into one-step and two-step methods (Table 7). Both rely on indirect organogenesis via callus formation, but they differ in hormone application: the one-step method uses a single hormone combination for both callus induction and shoot differentiation, whereas the two-step method employs distinct hormone formulations for each stage (Table 8). The one-step method is simpler and can produce plantlets more rapidly. Early reports suggested that the one-step method achieved higher shoot induction rates than the two-step method [112,113], although this may reflect incomplete understanding of ramie regeneration at the time, as the hormone formulation used for shoot induction in the two-step method was not fully optimized. Because the authors did not provide the exact hormone composition used for comparison, this claim cannot be fully evaluated. Conceptually, explants have different hormonal requirements at different developmental stages, and the two-step method allows more precise regulation. Historically, the one-step method was applied earlier in ramie transformation [108,109], and some studies have reported low transformation efficiency when using it [119]. Overall, neither method is inherently superior; the key determinant is the hormone formulation. When using a one-step protocol, if shoot induction consistently fails, it may be beneficial to adopt the hormone combinations used for shoot induction in two-step protocols.

Regarding hormones, thidiazuron (TDZ) should be considered. TDZ is a cytokinin-like growth regulator that plays an important role in ramie regeneration during genetic transformation. It modulates multiple metabolic pathways, enhances antioxidant capacity [124], supports the establishment and maintenance of stable callus cultures [125,126], and increases shoot regeneration frequency [111]. Recent studies frequently employ TDZ [20,21,114,117], and it forms the basis of efficient one-step regeneration systems [19,22,113,115,116,127], greatly accelerating ramie transformation workflows.

For antibiotics, when kanamycin is used as the selection agent, a concentration of 25–50 mg/L is commonly applied [19,108,109,117]. In addition, when *Agrobacterium* is involved, Agrobacterium-suppressing antibiotics must also be included in the culture medium. Cefotaxime (Cef) is preferred because it has minimal negative effects on ramie regeneration [111]; concentrations should not exceed 500 mg/L, with 200 mg/L being optimal [20]. Carbenicillin (Carb) is also commonly used in ramie studies. Some reports indicate that Carb inhibits shoot regeneration [111], whereas others show that it promotes cluster bud formation [110]. This discrepancy may arise because Carb degrades into phenylacetic acid (PAA), which exhibits auxin-like activity, whereas Cef does not [128]. Although one study reported that Cef increased the callus growth index (GI), its dose–response pattern differed markedly from that of Carb [129], and no similar effects of Cef have been observed in ramie to date; the underlying mechanism remains unclear. In cases where Cef alone cannot fully suppress *Agrobacterium*, a small amount of Carb may be added [119]. A practical approach is to apply a high antibiotic concentration initially to rapidly eliminate residual *Agrobacterium*, followed by a lower concentration in later stages to provide a gentler environment for explants [19,108,109].

#### 6.2.4. Chimerism and Purification

Many transgenic ramie plants exhibit mosaic GUS staining patterns, with some tissues showing positive signals and others remaining negative [19,21,108,117]. Ma et al. noted this phenomenon and suggested that the regenerated plants were likely chimeric [117].

In flax, chimerism has been specifically investigated. Transgenic flax plants also displayed uneven GUS staining, and the GUS-positive rate in their self-pollinated progeny was significantly lower than expected under Mendelian inheritance [130]. Two strategies have been proposed to address this issue:

(1) Self-pollination followed by screening for GUS-positive progeny;
(2) Vegetative propagation of tissues with low levels of chimerism, followed by segregation analysis of their progeny [130].

However, for ramie, its high genomic heterozygosity complicates sexual reproduction, and evaluating segregation ratios to distinguish homozygous from chimeric plants is time-consuming. If vegetative propagation is used to gradually recover non-chimeric sectors, fluorescent protein tagging offers an additional tool for identifying transformed tissues. Fluorescent proteins have been successfully applied in ramie [113,115] and are widely used in the studies discussed above. Introducing a fluorescent reporter allows non-destructive visualization of expression; tissues with strong fluorescence can be excised and regenerated through callus induction, potentially yielding plants with high genetic uniformity.

Another potential strategy for chimerism purification involves regeneration techniques derived from single cells, such as somatic embryogenesis and protoplast-based plant regeneration [131,132]. Although these methods have not yet been applied specifically for purifying transgenic ramie plants, both techniques have been successfully demonstrated in ramie and can regenerate whole plantlets. Because the regenerated plants originate from a single cell, the resulting individuals should be entirely transgenic or entirely wild-type, providing a theoretical route for eliminating chimerism.

#### 6.2.5. Prospects for Gene Editing

To date, no applications of CRISPR technology or gene-knockout studies have been reported in ramie. However, the successful establishment of CRISPR systems in other bast fiber crops demonstrates the feasibility of applying this technology in this kind of crops (Table 9). In 2022, CRISPR/Cas9 was first applied in flax by targeting LusCesA8, resulting in three edited seedlings with a survival rate of 33.33%. Sequencing revealed distinct mutation patterns among the edited plants: for example, line 8-4-1 carried only three adjacent base substitutions, whereas lines 8-4-3 and 8-3-5 exhibited more complex and widely distributed multi-site mutations [133]. Subsequently, more efficient CRISPR systems were developed in flax, and the performance of endogenous versus truncated U6 promoters for sgRNA transcription was evaluated. The endogenous U6 promoter showed slightly higher editing efficiency than the Arabidopsis AtU6-26 promoter [134,135].

In kenaf, CRISPR technology was initially applied in protoplasts to investigate miR394, which targets the LEAF CURLING RESPONSIVENESS (LCR) gene. However, the treated protoplasts exhibited poor viability and no regenerated seedlings were obtained [136]. Later, by targeting HcCLA1, albino seedlings were successfully produced, marking the establishment of a stable CRISPR/Cas9 editing system in kenaf [137]. In addition, CRISPR-based genome editing has also been achieved in other bast fiber crops such as hemp and jute in recent years [138,139].

Taken together, CRISPR represents a highly promising tool for functional validation of ramie genes, which could help address the current limitations in gene function research and accelerate the identification of causal genes relevant to fiber development and molecular breeding.

**Table 9:** Representative CRISPR/Cas-based genome-editing studies in major bast fiber crops.

Crop	Target Gene	Trait/Biological Process	Key Outcome	Ref.
flax	*LusCesA8*	Cellulose biosynthesis	Demonstrated the first successful CRISPR/Cas9 editing in flax and recovered three edited seedlings	[133]
flax	*LuPDS1*/*LuPDS2*	albino mutants; phytoene desaturase	Established a more efficient CRISPR/Cas9 editing system producing albino mutants	[134]
flax	*LuPDS1*	albino mutants; phytoene desaturase	Evaluated endogenous and truncated U6 promoters for editing efficiency	[135]
hemp	*CsPDS1*	albino mutants; phytoene desaturase	Established the first CRISPR/Cas9 editing system in hemp and identified highly regenerable cultivars	[138]
jute	*CcCLA1*	albino mutants; CLOROPLASTOS ALTERADOS 1	Developed the first stable CRISPR/Cas9 genome-editing system in jute	[139]
kenaf	miR394 precursor gene	miR394–LCR (LEAF CURLING RESPONSIVENESS) regulatory module	Achieved CRISPR/Cas9 editing in kenaf protoplasts, enabling functional analysis of the miR394–LCR module	[136]
kenaf	*HcCLA1*	albino mutants; CLOROPLASTOS ALTERADOS 1	Established a stable CRISPR/Cas9 editing system in kenaf and recovered three edited seedlings	[137]

## 7. Challenges, Knowledge Gaps, and Future Directions

Breeding technologies are shifting from traditional “empirical breeding” toward targeted and efficient “precision breeding,” encompassing approaches such as genomic selection, gene editing, de novo domestication, and intelligent design breeding. Elucidating the genetic and molecular basis of target traits is fundamental to achieving this transition. In ramie, substantial progress has been made in ramie genomics, and in understanding the genetic and molecular basis underlying fiber, thereby laying the foundation for molecular breeding aimed at fiber traits improvement. Nevertheless, several important gaps remain.

(1) Owing to strong inbreeding depression [59], developing near-isogenic lines (NILs) for fine mapping of genetic loci is difficult, which limits the validation of these loci. Coupled with this, inbreeding depression also presents challenges for purifying the genetic background of breeding parents, making it difficult to pyramid favorable alleles.
(2) Given that the genetic transformation system remains inefficient, most ramie genes must be functionally validated through heterologous expression in Arabidopsis, which hinders an in-depth understanding of the mechanisms underlying fiber trait formation. Consequently, the precise effects of genes in regulating fiber growth remain difficult to accurately assess.
(3) The low efficiency of the genetic transformation system remains a major bottleneck for transgenic breeding in ramie. Moreover, the absence of an established gene-editing system has hindered the application of advanced breeding technologies such as gene editing and de novo domestication.

In light of these limitations, future molecular breeding efforts in ramie should concentrate on the following aspects: First, develop SSR or Indel markers that co-segregate with loci/QTLs and genes regulating fiber traits, enabling the introgression of desirable alleles into cultivars via marker-assisted selection (MAS) to improve fiber yield and quality. Second, to enhance breeding efficiency, a genomic selection model should be constructed by integrating the genomic variation and phenotypic data now available for over 600 ramie germplasm accessions [18,45], enabling the selection of breeding parents based on genomic estimated breeding values (GEBV). Optimize the genetic transformation system to enable more efficient functional validation of genes and accurate evaluation of their effects. Fourth, establish an efficient gene-editing system for ramie, thereby facilitating the application of gene editing and de novo domestication technologies in breeding programs, ultimately enhancing fiber yield and quality.

## Figures and Tables

**Table 2:** Summary of key QTL and candidate genes associated with fiber traits.

Trait	QTL/Marker ID	Chr /LG	Genomic Position	Candidate Gene	Note	Ref.
fiber yield per stem	*qFY3*	LG3	-	-	-	[59]
fiber yield per stem	*qFY10*	LG10	-	-	-	[59]
stem length	*qSL10a*	LG10	-	-	-	[59]
stem number per plant	*qSN9*	LG9	-	-	-	[59]
bark thickness	*qBT2*	LG2	-	-	-	[59]
bark thickness	*qBT6*	LG6	-	-	-	[59]
fiber yield per stem	*qFY2*	LG2	74.5–76.8 cM	-	-	[60]
fiber yield per stem	*qFY4b*	LG4	102.7–104.1 cM	-	-	[60]
bark thickness	*qBT4a*	LG4	122.4–123.0 cM	*evm.model.scaffold7373.133_D1*	MYB transcription factor; 760 bp insertion; truncation	[60]
stem diameter	*qSD2b*	LG2	-	-	-	[61]
stem length	*qSL2*	LG2	-	-	in the same pleiotropic region as *qSD2b*	[61]
stem length	*qSL11b*	LG11	-	*whole_GLEAN_10018840*, *whole_GLEAN_10018848*, *whole_GLEAN_10018850*	three *DELLA* genes with sequence difference	[61]
fiber fineness	Ecor56873	Chr6	5,255,155 bp	-	-	[44]
fiber fineness	Ecor123653	Chr6	796,410 bp	-	-	[44]
fiber fineness	Ecor112647	Chr1	3,287,491 bp	-	-	[44]
fiber fineness	Ecor45552	Chr1	5,589,719 bp	-	-	[44]
fiber fineness	Ecor39620	Chr3	19,016,838 bp	-	-	[44]
fiber fineness, yield-related traits	-	-	-	-	no QTL ID provided, trait assignment shown only in the figure	[44]
fiber yield	*qFY5*	Chr5	-	*Bnt05G007931*	auxin-responsive gene; overlap with the selective sweeps	[10]
fiber yield	*qFY6*	Chr6	-	-	overlap with the selective sweeps	[10]
fiber yield per stem, stem diameter, bark weight	*SD11*/*BW11*/*FY11* (Cluster 10)	Chr11	135.2–147.0 cM	-	all QTLs displayed overdominance	[62]
fiber yield per stem, stem diameter, bark weight	*SD3*/*BW3*/*FY3* (Cluster 4)	Chr3	136.1–141.1 cM	*CL10581Contig1*	desiccation-related protein; positive selection gene	[62]
stem diameter, stem length, bark weight	*SD8*/*SL8*/*BW8* (Cluster 8)	Chr8	129.8–139.7 cM	*T3_Unigene_BMK.28528*	homeobox-leucine zipper protein; positive selection gene	[62]
fiber yield per stem, stem length	*SL9*/*FY9* (Cluster 9)	Chr9	133.5–136.7 cM	*CL16310Contig1*	ankyrin repeat-containing protein; positive selection gene	[62]
bark thickness	*BT1*	Chr1	97.3 cM	*CL12943Contig1*	WAT1-related protein; positive selection gene	[62]
crude fiber	*CF13*	Chr13	36.4 cM	*whole_GLEAN_10016511*	MYB transcription factor; overexpression in Arabidopsis increases fiber cells	[63]
lignin content	*qLC7*	LG7	-	*whole_GLEAN_10021050*	Transcription factor MYB106	[36]
lignin content	*qLC10*	LG10	-	*whole_GLEAN_10026962*	Laccase-17	[36]
lignin content	*qLC13*	LG13	-	*whole_GLEAN_10009464*	Omega-hydroxypalmitate O-feruloyl transferase	[36]

**Table 3:** Summary of key GWAS, SSR association and candidate genes associated with fiber traits.

Trait	SNP/Marker ID	Chr	Genomic Position	Candidate Gene	Note	Ref.
stem diameter	RAM290	-	-	-	-	[64]
skin thickness	RAM200	-	-	-	-	[64]
skin thickness	RAM141	-	-	-	-	[64]
skin thickness	RAM144	-	-	-	-	[64]
ramet number	Marker20170–64	-	-	*Bn23049*, *Bn23037*, *Bn23055*, *Bn23053*, *Bn23057*, *Bn23041*	associated with ramet number; validated in F1 population	[65]
ramet number	Marker142939–43	-	-	-	detected across three stages	[65]
fiber fineness	PHNS01005842.1:2705313	-	-	*whole_GLEAN_10029622*, *whole_GLEAN_10029638*	qPCR validated; cotton fiber–expressed gene; homeobox protein ATH1	[58]
fiber percentage	PHNS01011408.1:355775	-	-	*Whole_GLEAN_10007759*	WAT1-related protein homolog	[58]
fiber percentage	PHNS01001992.1:258552	-	-	*whole_GLEAN_10025406*	auxin-responsive protein IAA13 homolog	[58]
fiber percentage	PHNS01010418.1:933907	-	-	*whole_GLEAN_10017958*	NAC domain-containing protein	[58]
skin thickness	PHNS01006314.1:1911874	-	-	*Whole_GLEAN_10021913*	BES1/EZR1 transcription factor homolog, BES1 target at MYB30 in *Arabidopsis*	[58]
fiber fineness	9_3323163	Chr9	3,223,163–3,423,163 bp	*Bng.009591*	1-aminocyclopropane-1-carboxylate oxidase homolog	[45]
fiber fineness	11_17461618	Chr11	17,361,618–17,561,618 bp	*Bng.008687*	cellulose synthase-like protein E1	[45]
fiber fineness	4_23390231	Chr4	23,290,231–23,490,231 bp	*Bng.000585*	MYB30 transcription factor	[45]
fiber fineness	1_1992520	Chr1	1,892,520–2,092,520 bp	*Bng.076290*	MYB transcription factor APL	[45]
fiber fineness	6_16308180	Chr6	16,208,180–16,408,180 bp	*Bng.042170*	kinesin-like protein KIN-7D	[45]
fiber fineness	8_11456538	Chr8	11,356,538–11,556,538 bp	*Bng.086916*	KINESIN LIGHT CHAIN-RELATED 1 Protein	[45]
bark thickness	S7_19790489	Chr7	-	*Bng.064573*	Alpha-1,4-glucan-protein synthase [UDP-forming] 2	[45]
bark thickness, stem diameter	S8:17884159, S7:17881893	-	-	*Bng.064280* (*BnNAC29*)	causal SNP located in the promoter; overexpression in *Arabidopsis* increases fibers cells	[45]
bark thickness	-	-	-	*Bng.064534*	MYB88 transcription factor	[45]
bark thickness	-	-	-	*Bng.064735*	expansin protein A1	[45]
stem diameter	S8_18255615	Chr8	-	*Bng.072557* (*BnVIT1*)	vacuolar iron transporter 1	[45]
bark thickness	-	Chr10	-	*Bng.062584* (*BnAEP*)	aldose 1-epimerase	[45]
fiber fineness	S9_5291968	Chr9	5,191,968–5,391,968 bp	*Bng.009770*	putative pectinesterase 29 homolog; underwent positive selection	[45]
bark weight	-	Chr10	14,843,234 bp	*Bnt10G015742*	strongest signal associated with fiber yield in this study	[18]
Bark thickness	-	Chr9	17,322,051 bp	*Bnt09G014694*	SBP-box transcription factor gene; signal associated on coding region	[18]
fiber output ratio	-	Chr3	18,319,491 bp	*Bnt03G004997*	NAC domain-containing protein gene; signal associated on promoter; overexpression in *Arabidopsis* increases fiber number	[18]
fiber output ratio	-	Chr10	5,882,131 bp	*Bnt10G014913*	COBRA-like protein gene; signal associated on downstream	[18]
fiber yield, bark weight	-	Chr10	15,536,000 bp	*Bnt10G015830*	peroxidase gene; signal associated on coding region; certain genotypes with frameshift linked to higher bark and fiber yield	[18]
bark weight, fiber yield	-	Chr14	6,934,389 bp	*Bnt14G019616*	pectin methylesterase; signal associated on coding region; overexpression in *Arabidopsis* decreases fiber content	[18]
hemicellulose	SNP: Maker75072385	Chr1	4,825,385 bp	*Maker00075072*	NAC domain-containing protein	[13]
hemicellulose	Maker76265070	Chr1	2,888,070 bp	*Maker00076265*	cytochrome P450 90A1	[13]
hemicellulose	Maker75027661	Chr1	4,418,661 bp	*Maker00075027*	Ethylene-responsive transcription factor	[13]
hemicellulose	Maker86604278	Chr8	12,912,278 bp	*Maker00086604*	Auxin response factor	[13]
pectin	Maker09488307	Chr9	5,462,307 bp	*Maker00009488*	probable E3 ubiquitin-protein ligase	[13]
pectin	Maker83008111/Maker83008114	Chr13	9,507,111 bp, 9,507,114 bp	*Maker00083008*	protein NRT1/PTR FAMILY 5.10-like	[13]
hydrotrope	Maker00423622	Chr4	22,100,622 bp	*Maker00000423*	6-phosphogluconate dehydrogenase	[13]

**Table 5:** Functionally validated fiber-related genes in ramie (*Boehmeria nivea*) and their phenotypic effects. All genes listed are those that have been functionally validated to date. Functional validation was performed either in ramie itself or in heterologous systems such as Arabidopsis thaliana.

Gene	Function Summary	Phenotype Description	Species Used for Functional Validation	Ref.
*Marker00009591*	ACC oxidase; may regulate ethylene biosynthesis to influence cell elongation and fiber fineness	Increased fiber fineness	Ramie	[46]
*Bnt05G007257*	Homolog of *SND2*; NAC transcription factor regulating secondary wall biosynthesis	Increased bast and xylem fiber diameter	*Arabidopsis*	[47]
*BnNAC29*	NAC transcription factor promoting cell division and differentiation	Increased bast fiber cell size and number	*Arabidopsis*	[45]
*Bnt03G004997*	Homolog of *VND4*/*5*; NAC transcription factor regulating secondary wall biosynthesis	Increased fiber number	*Arabidopsis*	[18]
*Bnt14G019616*	Pectin methylesterase; key enzyme for catalyzing demethoxylation of pectin	Decreased fiber content	*Arabidopsis*	[18]
*Bnt07G011994*	Homolog of *KNAT7*; KNOX transcription factor regulating fiber growth	Suppressed xylem fiber growth	*Arabidopsis*	[17]
*whole_GLEAN_10029667*	Homolog of *KNAT1*; KNOX transcription factor regulating fiber growth	Suppressed fiber growth	*Arabidopsis*	[90]
*Bnt08G012573*	Homolog of *NST1*/*2*; NAC transcription factor regulating secondary wall biosynthesis	Increased bast fiber number	*Arabidopsis*	[10]
*whole_GLEAN_10015497*	Homolog of *MYB51*; transcription factor promoting fiber development	Increased bast and xylem fiber number and cell wall thickening	*Arabidopsis*	[87]
*whole_GLEAN_10024150*	Homolog of *RWA3*; promoting fiber development	Increased xylem fiber number and cell wall thickening	*Arabidopsi* *s*	[87]
*BntWG10016451*	*MYB51* transcription factor promoting fiber development	Increased xylem fiber number and cell wall thickening	*Arabidopsi* *s*	[88]
*whole_GLEAN_10016511*	MYB transcription factor *KAN2*; regulating vascular tissue formation	Increased fiber cells	*Arabidopsi* *s*	[63]

**Table 6:** Summary of six ramie genome assemblies.

Item	*B. nivea* var. *tenacissima*	Zhongsizhu 1	Zhongzhu 1 (Chen et al. [44])	Zhongzhu 1 (Liu et al. [15])	Zhongzhu 1 (Luan et al. [64])	HZS10	1380
Size of the assembly (Mb)	270.21	266.60	268.24	335.58	341.19	294	344
Scaffold number	135	330	522	293,578	10,339	-	-
Scaffold N50 (Mb)	19.55	17.80	17.611	0.0423	1.126	21.64	-
Contig number	450	657	522	344,363	-	-	14
Contig N50 (Mb)	10.51	2.33	1.369	0.0037	0.0226	3.42	17.54
Annotated protein-coding genes	20,693	19,498	27,664	42,463	30,237	22,312	25,853
TE proportion (%)	45.5	44.3	45.51	-	46.3	54.85	61.19
LTR assembly index	23.38	19.28	14.47	-	-	-	21.74
Complete BUSCOs (%)	96.9	96.9	98.10	-	90.5	95.8	99.8
Complete and single-copy BUSCOs	93.9	94.2	94.3	-	84.2	94.4	98.6
Complete and duplicated copy BUSCOs	3.0	2.7	3.8	-	6.3	1.4	1.2
Fragmented (%)	1.3	1.7	0.7	-	4.9	-	-
Missing (%)	1.8	1.4	1.2	-	4.6	-	-

**Table 7:** Cultivars, explant origins (sexual vs. asexual), regeneration modes (one-step vs. two-step), and Agrobacterium strains used in representative studies of Agrobacterium-mediated genetic transformation in ramie.

Year	Cultivar(s)	Explant Origin	Regeneration Mode	Agrobacterium Strain	Ref.
1993	BRA000027	Asexual (clonal)	One-step	EHA101	[108]
2000	Xiangmang NO.3	Sexual (seed-derived)	One-step	EHA105	[110]
2005	Xiangzhu NO.3, Luzhuqing, Qingma, Yuanma	Asexual (clonal)	One-step	LBA4404	[118]
2006	Xiangzhu NO.2	Sexual (seed-derived)	One-step	LBA4404, EHA105	[112]
2010	Zhongzhu NO.1, Zhongsizhu NO.1, NC01	Asexual (clonal)	Two-step	LBA4404	[117]
2014	Huazhu NO.5	Asexual (clonal)	One-step	LBA4404	[19]
2025	Zhongzhu NO.1	Asexual (clonal)	Two-step	LBA4404	[20]

**Table 8:** Explants, culture media, and hormone combinations used in representative studies of Agrobacterium-mediated genetic transformation in ramie.

Year	Explant	Callus Induction Medium (mg/L)	Shoot Induction Medium (mg/L)	Ref.
1993	Leaf disc	—	B5 + 6-BA 2.0 + IAA 0.2 or NAA 0.02 *	[108]
2000	Hypocotyl	—	½ MS + 6-BA 2.0 + IAA 0.5 + AgNO_3_ 1.0 *	[110]
2005	Leaf disc	—	½ MS + 6-BA 3.0–4.0 or KT 3.0 + NAA 0.01 *	[118]
2006	Hypocotyl	—	MS + 6-BA 2.0 + NAA 0.1 *	[112]
2010	Cotyledon, Hypocotyl	MS + 6-BA 0.25 + IAA 0.12	MS + TDZ 0.01 + IAA 0.12 + GA_3_ 0.12	[117]
2014	Leaf midrib	—	MS + TDZ 0.2 + 2,4-D 0.04 *	[19]
2025	Leaf disc	½ MS + TDZ 0.05 + 2,4-D 0.03 + IAA 0.01	½ MS + TDZ 0.5 + 2,4-D 0.02 + IAA 0.03	[20]

* One-step regeneration.

## Data Availability

No new data were created or analyzed in this study. Data sharing is not applicable to this article.
